# Efficiency of red cell distribution width in predicting severity and mortality of patients with acute pancreatitis: A protocol for systematic review and meta-analysis

**DOI:** 10.1097/MD.0000000000024658

**Published:** 2021-02-12

**Authors:** Tao Cheng, Bo-Fu Liu, Tian-Yong Han, Pan Pan, Jun-Zhao Liu, Haifang Yu

**Affiliations:** a Emergency Department; b Laboratory of Emergency Medicine, West China Hospital, Sichuan University; c Disaster Medical Center, Sichuan University, Chengdu, Sichuan, China.

**Keywords:** acute pancreatitis, meta-analysis, mortality, red cell distribution width, severity

## Abstract

**Background:**

Previous studies have showed that red cell distribution width (RDW) may be an inflammatory status, and it may be used to predict prognosis of acute pancreatitis (AP). However, there are no systematic reviews for the evidence, and the association between RDW and AP is still not completely understood. Therefore, we will undertake a systematic review of the literature to summarize previous evidence regarding this topic, in order to clarify the value of RDW predicting prognosis of patients with AP.

**Methods:**

We will search EMBASE, Web of Knowledge, PubMed, ClinicalTrials.gov and Cochrane Library from their inception to Mar 2021 to retrieve relevant studies. Two authors independently judged study eligibility and extracted data. Heterogeneity will be examined by computing the Q statistic and I^2^ statistic.

**Results:**

This study proved the Efficiency of RDW in predicting mortality and severity of patients with AP. And provided easy method for clinical evaluation for AP patients.

**Conclusions:**

The findings of this systematic review will show the value of RDW predicting prognosis of patients with AP.

**Ethics and dissemination:**

Ethical approval is unnecessary as this protocol is only for systematic review and does not involve privacy data. The findings of this study will be disseminated electronically through a peer-review publication or presented at a relevant conference.

## 1 Introduction

Acute pancreatitis is a sudden inflammatory process in the pancreas with variable involvement of nearby organs or other organ systems,^[1–3]^ part of one of the leading causes of hospitalization among gastrointestinal diseases.^[4]^ Based on the revised Atlanta classification, the severity of AP was classified by 3 degrees: mild acute pancreatitis (MAP), moderately severe acute pancreatitis (MSAP), and severe acute pancreatitis (SAP).^[5]^ The Rates of mortality following an attack of acute pancreatitis are between 2% and 20%, according to severity.^[6–8]^ Patients with MAP often recover spontaneously within a few days, but the management of patients with MSAP or SAP remains a challenge.^[6,8–10]^ Therefore, it is necessary to predict the severity of AP during the early stages, for it is critical for determining early triage, aggressive resuscitation, and improving clinical outcomes of AP in high-risk patients.

Currently, there are various scoring systems used to predict the severity and mortality of AP, such as Ranson criteria,^[11]^ Glasgow-Imrie score,^[12]^ Acute Physiology and Chronic Health Evaluation II (APACHE II),^[13]^ Multiple Organ System Score (MOSS)^[14]^ and Balthazar grade.^[15,16]^ However, the scoring systems involve many tests and are cumbersome to operate. They are not convenient for clinical practice. In this connection, it is important to identify a simple, easy, and sensitive marker to predict the severity and mortality of AP.

Red cell distribution width (RDW) is a routine parameter of the complete blood count test, described as simple, easy, inexpensive and quantitative that measures the size heterogeneity of peripheral red blood cell (RBC).^[17–19]^ AP is an inflammatory disease which caused by abnormal activation and release of digestive enzymes and it is characterized by increased cytokine release and oxidative stress.^[20]^ It is well established that the severity of AP ultimately depends on the intensity of systemic inflammatory response, RDW may be an inflammatory status, such as C-reactive protein, interleukin 6, and fibrinogen, and it may be used to predict severity and mortality in patients with AP.^[21–25]^ However, there are no systematic reviews for the evidence, and the association between RDW and AP is still not completely understood. Therefore, we will undertake a systematic review of the literature to summarize whether RDW is a simple, easy, and sensitive indicator to predict the prognosis of patients with AP.

## 2 Methods and analysis

### 2.1 Registration

This meta-analysis protocol is based on the Preferred Reporting Items for Systematic Reviews and meta-analysis Protocols (PRISMA-P) statement guidelines.^[24]^ The PRISMA-P checklist for the protocol is provided in the PRISMAP-checklist. This protocol has been registered on International Prospective Register of Systematic Reviews database. The registration number was INPLASY202110042.

### 2.2 Eligibility criteria

The inclusion criteria for the study will include:

(1) studies with patient age ≥18 years old, a minimum hospital stay of 24 hour and a diagnosis of AP;
(2) conference abstracts were only included when they provided adequate relevant information for assessment;
(3) the RDW was used for the prediction of severity or mortality of patients with AP.

Exclusion criteria will include: age <18 years old, patients with chronic pancreatitis or pancreas carcinoma and patients with incomplete data and the presence of underlying factors that could change RDW, such as infectious or immunosuppressive conditions/therapy, active malignancy, late stage of liver cirrhosis, active tuberculosis, refractory heart failure, chronic use of erythropoietin, recent transfusion history, pregnancy or trauma.

### 2.3 Searching strategy

**Figure 1:**
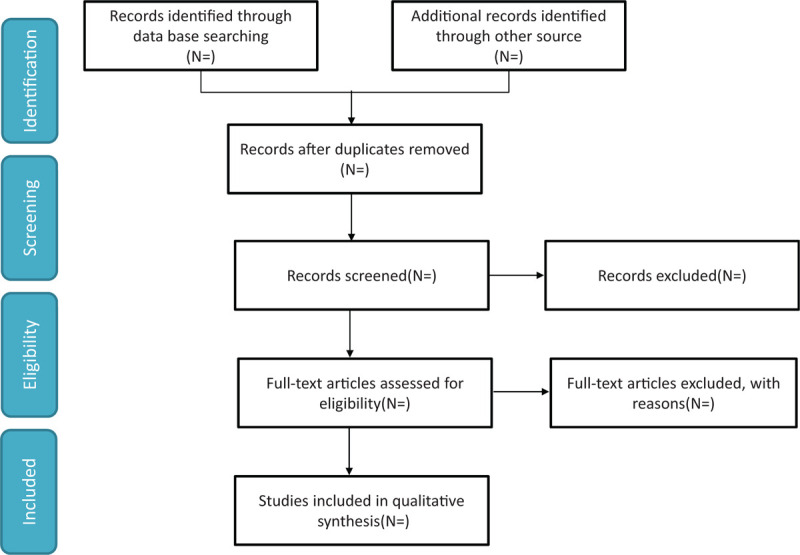
A flow diagram demonstrating the search strategy and study selection process for this study.

We will search the EMBASE, Web of Knowledge, PubMed, ClinicalTrials.gov and Cochrane Library from inception to Mar 2021 to retrieve relevant studies using the search strategy: (“red cell distribution width” OR “RDW” OR “red blood distribution width” OR “Erythrocyte Indices” OR anisocytosis) AND (pancreatitis OR pancreatitides). No language restrictions will be applied. We will also search citations of relevant primary and review. Authors of abstract in the meeting will be further searched in PubMed for potential full articles. To minimize the risk of publication bias, we will conduct a comprehensive search that included strategies to find published and unpublished studies. The research summary of the screening flow chart is shown in Figure 1.

### 2.4 Data extraction and risk of bias

Two reviewers will be employed the searching strategy respectively, by reading the papers and scoring them according to the QUADAS-2 checklist^[26]^ and Newcastle–Ottawa Quality Assessment Scale;^[27]^ disagreement will be settled by a third opinion. Important information will be abstracted from the included articles in a standardized form by two reviewers. Important information includ the name of the first author, publication year, publication country, type of study, study population, sample size, defined criteria of SAP, outcomes studied (severity and mortality rate) and RDW. Risk of bias assessment will be carried out according to the Newcastle-Ottawa Scale (NOS) to rate the internal validity of the individual studies, and funnel plots will be constructed to assess the risk of publication bias.

### 2.5 Statistical analysis

All pairwise meta-analytic calculations will be performed with Review Manager software (RevMan) version 5.3 (Cochrane Collaboration). Heterogeneity will be examined by computing the Q statistic and I^2^ statistic, and presence of reporting bias by visual inspection of funnel plots. Statistical significance was considered when the *P* value <.05.

## 3 Discussion

It is well known that severe AP cases are often associated with severe complications and high mortality.^[28]^ However, MAP cases do not need long hospitalization, and even can be treated out of hospital to recover quickly, which could reduce the proportion of hospitalizations and therefore save more costs. Over years, several scoring systems, such as Ranson criteria,^[11]^ APACHE II,^[13]^ and Balthazar grade,^[15,16]^ have been well-established to predict the severity and mortality of AP. However, both of them have significant weaknesses. For example, the Ranson criteria requires 48 hours to complete, which will miss the potentially valuable early treatment. RDW, as a simple, easy and inexpensive marker, has been reported that it may be used to predict severity and mortality in patients with AP,^[22–25]^ but the conclusion is controversial. To identify whether RDW can be used to predict the severity and mortality of AP, we conducted this meta-analysis.

Therefore, there is an urgent requirement to make a systematic review of studies on the prognostic significance of RDW for AP. This article is a protocol of our systematic review, which presented the detailed description of review implement. The results of our review will be reported strictly following the PRISMA criteria. By integrating the data from previous articles, this review will objectively reveal the relationship between the expression of RDW and prognosis of patients with AP, and clarify the value of RDW predicting prognosis of patients with AP.

## Acknowledgments

The authors acknowledge the participants and their families for taking part in the study.

## Author contributions

**Conceptualization:** Tao Cheng, Bofu Liu.

**Data curation:** Tao Cheng, Tian-Yong Han, Bo-Fu Liu, Pan Pan.

**Formal analysis:** Tao Cheng, Jun-Zhao Liu, Tian-Yong Han, Pan Pan.

**Funding acquisition:** Haifang Yu.

**Investigation:** Tao Cheng, Bofu Liu, Tian-Yong Han, Jun-Zhao Liu.

**Methodology:** Tao Cheng, Bofu Liu, Tian-Yong Han, Jun-Zhao Liu.

**Project administration:** Haifang Yu.

**Resources:** Jun-Zhao Liu, Pan Pan.

**Software:** Tao Cheng, Bofu Liu.

**Supervision:** Haifang Yu.

**Validation:** Tao Cheng, Bofu Liu.

**Visualization:** Bofu Liu, Tao Cheng.

**Writing – original draft:** Bofu Liu, Tao Cheng.

**Writing – review & editing:** Haifang Yu.

## Abbreviations

Abbreviations: APACHE II = acute physiology and chronic health evaluation II, CI = confidence interval, MAP = mild acute pancreatitis, MOSS = multiple organ system score, NOS = Newcastle-Ottawa Scale, RBC = red blood cell, RDW = red cell distribution width, SAP = severe acute pancreatitis.
